# New variants in ACE gene region in 10 Cavalier King Charles Spaniel dogs with mild and severe myxomatous mitral valve disease – preliminary study

**DOI:** 10.1016/j.vas.2026.100769

**Published:** 2026-07-13

**Authors:** Maksymilian Lewicki, Sylwia Górczyńska-Kosiorz, Piotr Frydrychowski, Justyn Gach, Zuzanna Wojtczak, Jacek Cymbryłowicz, Agnieszka Noszczyk-Nowak

**Affiliations:** a Department of Internal Medicine and Clinic of Diseases of Horses, Dogs and Cats, Wrocław University of Environmental and Life Sciences, Grunwaldzki Sq. 47, 50-366, Wrocław, Poland; b Department of Internal Medicine, Diabetology and Nephrology, Faculty of Medical Sciences in Zabrze, Medical University of Silesia, 40-055, Katowice, Poland; c Gliwicka Przychodnia Weterynaryjna, Toszecka 19 Str, Gliwice, 44-100, Poland

**Keywords:** MMVD, CKCS, ACE, SNP, dog, gene

## Abstract

Myxomatous mitral valve disease is the most common acquired cardiac disorder in dogs, with particularly high prevalence and early onset in Cavalier King Charles Spaniels. Activation of the renin-angiotensin-aldosterone system plays a key role in disease progression, and genetic variation in the angiotensin-converting enzyme gene may influence disease severity and therapeutic response. The aim of this study was to characterize angiotensin-converting enzyme gene variation in a Polish Cavalier King Charles Spaniel population with mild and severe myxomatous mitral valve disease using targeted next-generation sequencing, with emphasis on the rs850683722 polymorphism.

Ten client-owned dogs diagnosed with myxomatous mitral valve disease were prospectively enrolled and classified according to American College of Veterinary Internal Medicine guidelines into mild (stage B1) and severe (stage C/D) groups. Dogs with comorbidities or receiving medications affecting the renin-angiotensin-aldosterone system were excluded. Genomic DNA isolated from peripheral blood underwent next-generation sequencing to cover coding exons and adjacent intronic regions of the angiotensin-converting enzyme gene. Variant detection and annotation were performed using standard bioinformatic methods.

A total of 188 variants were identified, of which 95,2 % were intronic. Coding variants were rare and included one missense substitution, one in-frame insertion, and three synonymous changes. Fourteen variants were novel. The rs850683722 polymorphism was present in both groups without clear association with disease stage. No variant showed a strong relationship with disease severity.

Overall, angiotensin-converting enzyme gene variation was dominated by non-coding polymorphisms with potential regulatory effects, suggesting a modifying rather than causative role in disease progression.

## 1 Introduction

Myxomatous mitral valve disease (MMVD) is the most common acquired cardiac disorder in dogs and represents the leading cause of congestive heart failure in small- and toy-breed dog populations (Atkins et al., 2009, Keene et al., 2019, Parker and Kilroy-Glynn, 2012, Svensson et al., 2024, Borgarelli et al., 2008, Vezzosi et al., 2025). One breed particularly affected by myxomatous mitral valve disease is the Cavalier King Charles Spaniel (CKCS). Epidemiological studies indicate that nearly all CKCS develop echocardiographic or auscultatory signs of MMVD by advanced age, with a substantial proportion exhibiting murmurs before five years of age (Prieto Ramos et al., 2021, Lewis et al., 2011, Bagardi et al., 2020, Beardow and Buchanan, 1993, Summers et al., 2015, Sudunagunta et al., 2019). The severe and predictable disease burden in this breed makes CKCS a valuable population for investigating the genetic basis of MMVD susceptibility, progression rate, and therapeutic response.

Despite extensive clinical and pathomorphological characterization of MMVD, the molecular and genetic mechanisms underlying disease heterogeneity remain incompletely understood. Numerous studies have demonstrated that MMVD pathogenesis involves progressive extracellular matrix (ECM) remodeling, activation of valvular interstitial cells (VICs), dysregulation of transforming growth factor-β (TGF-β) signaling, oxidative stress, and neurohormonal activation, particularly of the renin-angiotensin-aldosterone system (RAAS) (Fox, 2012, Druzhaeva et al., 2022, Li et al., 2019, Boland et al., 2018, Oyama et al., 2020, Markby et al., 2017, Gach et al., 2025, Lu et al., 2016). However, marked inter-individual variability in disease course within genetically predisposed breeds suggests the presence of additional genetic modifiers that influence neurohormonal regulation and tissue remodeling processes (Lewicki et al., 2025, Mellanby et al., 2013, French et al., 2012, Purevjav et al., 2010, Perrot et al., 2016, Maiellaro-Rafferty et al., 2013, Axelsson et al., 2021, Mead et al., 2022).

The renin-angiotensin-aldosterone system (RAAS) is an important modifier of disease progression in canine myxomatous mitral valve disease (MMVD), particularly after chronic mitral regurgitation has produced progressive left atrial and ventricular volume overload. Reduced effective cardiac forward output and altered renal perfusion may activate the classical RAAS pathway, increasing angiotensin II and aldosterone signalling. Although initially compensatory through maintenance of arterial pressure and intravascular volume, persistent RAAS activation becomes maladaptive by promoting vasoconstriction, sodium and water retention, increased preload and afterload, sympathetic facilitation, endothelial dysfunction, oxidative stress, inflammation, and myocardial and interstitial fibrosis. These effects may amplify eccentric cardiac remodelling and contribute to the transition from compensated MMVD to congestive heart failure. Importantly, RAAS involvement in MMVD is not limited to systemic circulating activation. Experimental and clinical data suggest that tissue-level RAAS modulation may occur even when plasma RAAS markers are not clearly increased, and recent work has also highlighted alterations in both classical and alternative renin–angiotensin pathways in dogs with preclinical stage B2 MMVD (Ames et al., 2017, Hammond et al., 2023). Aldosterone is particularly relevant because, beyond its renal effects, it may promote cardiovascular fibrosis and remodelling; moreover, aldosterone breakthrough has been documented in dogs with naturally occurring MMVD receiving ACE-inhibitor therapy, supporting the rationale for combined RAAS blockade in selected patients.

Genes encoding RAAS components have therefore emerged as plausible candidates influencing both disease expression and treatment efficacy in MMVD. Among these, angiotensin-converting enzyme (ACE) plays a central role by catalyzing the conversion of angiotensin I to angiotensin II and regulating aldosterone synthesis. In the current ROS_Cfam_1.0 assembly, the canine ACE gene is located on chromosome 9 at 13,160,704-13,200,795, spans 40,092 bp, and the canonical transcript ENSCAFT00845032546.1 contains 26 exons.

Variation in ACE activity may directly affect hemodynamic load, myocardial remodeling, and fibrotic processes associated with chronic mitral regurgitation. This association with mitral valve disease has already been described in humans, however, research searching for genes responsible for human MMVD in dogs have not yielded positive results and candidate genes associated with human myxomatous mitral valve disease have not shown clear associations with familial MMVD in dogs. (Meurs et al., 2018). Previous investigations by Meurs et al have reported polymorphisms within canine RAAS-related genes, including ACE, with potential associations to cardiovascular phenotypes and biomarker profiles in dogs. Subsequent population studies confirmed the presence of specific ACE gene variants in multiple dog breeds and emphasized breed-related frequency differences. However, the clinical significance of these polymorphisms, particularly in breeds strongly predisposed to MMVD such as CKCS, remains unclear (Ames et al., 2017, Hammond et al., 2023, Meurs et al., 2018, Meurs et al., 2018, Meurs et al., 2015, Adin et al., 2021, Meurs et al., 2017, Adin et al., 2020).

One variant of particular interest is rs850683722, which has generated inconsistent findings across studies regarding its prevalence and potential functional relevance (Meurs et al., 2018, Meurs et al., 2018, Meurs et al., 2015, Adin et al., 2021, Meurs et al., 2017, Adin et al., 2020). While the effect of the above-mentioned variant on the activity of angiotensin-converting enzyme has been confirmed, studies on the effectiveness of therapy based on RAAS blockade using, among others, ACE inhibitors remain inconsistent. These discrepancies may stem from limited breed-specific cohorts, lack of disease severity stratification, or insufficient integration of biochemical phenotype data with genetic profiling.

In CKCS populations specifically, targeted analyses addressing ACE gene variants in relation to MMVD severity remain scarce. Most available reports have either analyzed mixed-breed cohorts or focused primarily on variant frequency without integrating detailed clinical staging or endocrine biomarker assessment. Therefore, it remains unclear whether ACE polymorphisms - particularly rs850683722 - contribute to individual differences in MMVD clinical expression or hormonal activation patterns within this genetically susceptible breed.

The objective of the present study was to evaluate the distribution of ACE gene variants in Polish population of CKCS dogs with clinically mild versus severe MMVD using next-generation sequencing (NGS) and to investigate prevalence of rs850683722 variant in studied population and its relation to the severity of the disease.

## 2 Materials and methods

### 2.1 Study population and clinical classification

The inclusion criteria for the study were: breed – CKCS, age over 6 years, lack of kinship between individuals and diagnosis of MMVD according to the American College of Veterinary Internal Medicine (ACVIM) classification (Atkins et al., 2009, Keene et al., 2019, Wess et al., 2020, Coffman et al., 2021, Borgarelli et al., 2020). The control group was to consist of dogs in stage A. If it was not possible to recruit dogs over 6 years of age in stage A, the control group would consist of dogs in stage B1 and comparison would be carried between the dogs in subclinical and clinical stages. The study was conducted at the Department of Internal Medicine and Clinic of Diseases of Horses, Dogs and Cats, Wrocław University of Environmental and Life Sciences, Grunwaldzki Sq. 47, 50-366 Wrocław, Poland, and “GLIWICKA PRZYCHODNIA WETERYNARYJNA” Toszecka 19 str. Gliwice, Poland. Written informed owner consent was obtained prior to inclusion in the study. Dogs with congenital heart defects and other cardiovascular disorders such as dilated cardiomyopathy phenotype or heart tumors, systemic conditions potentially influencing RAAS activity (e.g., renal disease, endocrine disorders, neoplasia), or receiving medications known to directly affect renin-angiotensin-aldosterone system (e.g., ACE inhibitors, mineralocorticoid receptor antagonists, glucocorticosteroids) were excluded from the study.

Dogs underwent standardized cardiological evaluation including physical examination, cardiac auscultation, transthoracic echocardiography (2D, M-mode, and Doppler imaging) (Philips Ultrasound Inc. EPIQ Elite, USA; GE Vingmed Ultrasound AS, VIVID S70n, Norway), resting electrocardiography (BTL Industries Ltd., BTL-08 MT Plus, UK) and arterial blood pressure measurement using the doppler method (Parks medical electronics inc. model 811-B, USA). MMVD diagnosis was based on the ACVIM consensus classification. For the purposes of genetic association analysis, dogs were allocated into two clinical phenotype groups:

Mild MMVD group: dogs over 6 years of age, ACVIM stage B1.

Severe MMVD group: dogs over 6 years of age, ACVIM stage C, or D

### 2.2 DNA sampling and extraction

Peripheral whole blood samples (EDTA, volume: 2 mL) were collected from each dog for genomic analysis using vacuum blood collection system. Genomic DNA was extracted using a commercially available extraction kit (MagCore® Genomic DNA Whole Blood Kit) following the manufacturer’s protocol. DNA quantity and purity were assessed by spectrophotometry (DeNovix DS-11), and samples not meeting quality thresholds (A260/280 ratio 1.6–2.0) were reprocessed.

### 2.3 Next-generation sequencing

Library preparation and sequencing were performed on an Illumina NovaSeq series instrument in 2 × 150 nt mode with 25-30x coverage. Raw reads (FASTQ) were subjected to initial quality control and bioinformatics processing according to standard NGS data analysis practices.

Reads were trimmed to remove adapters and low-quality ends using Cutadapt v2.8.

Trimmed reads were mapped to the dog reference genome (ROS_Cfam_1.0, GCF_014441545.1) using the Burrows-Wheeler Aligner algorithm (BWA-MEM v0.7.15-r1140).

The resulting BAM files were sorted using Picard v2.20.4. Duplicate reads in the BAM file were located and marked using the MarkDuplicates module from the Picard package.

Base quality score recalibration (BQSR) was performed using the Genome Analysis Toolkit (GATK) v4.2.2.0.

Detection of SNP and indel variants within the ACE gene (as well as detection of variants located up to 1500 nucleotides upstream of the ACE gene) was performed using the HaplotypeCaller tool from the GATK v4.2.2.0 package. The analysis was conducted according to the recommended GATK Best Practices workflow.

The resulting VCF files were normalized using bcftools v1.6, which included the decomposition of multiallelic variants and left-alignment of indels relative to the reference genome.

The final set of variants was functionally annotated using the Variant Effect Predictor (VEP) tool - ROS_Cfam_1.0, which allowed for the determination of the potential impact of the variants on the genes.

The analyzed region included the complete ACE gene sequence according to ROS_Cfam_1.0, together with up to 1.5 kb upstream of the gene.

### 2.4 Statistical analysis

Fisher’s exact tests were performed to assess the distribution of all identified variants between dogs with mild and severe MMVD and also in relation to gender. Detected variants were analysed in two complementary models: an allelic model comparing reference and alternative allele counts, and a carrier model comparing dogs carrying at least one alternative allele with non-carriers. For variants with several alternative variants, the simplified "any alt" model was used. Statistical analysis was performed using Graphpad Prism v5.03.

### 2.5 Ethics approval statement

In accordance with the Experiments on Animals Act from January 15th 2015 (Journal of Laws of the Republic of Poland, 2015, item. 266), concerning the welfare of the animals used for research or teaching purposes, the restrictions defined in the law shall not apply to:

(1). clinical veterinary practices as defined by the Act from December 18th 2003 concerning veterinary practices (Journal of Laws from 2004, No. 11, item 95 as amended in item 3), as well as agricultural activity, raising and breeding livestock according to the Animal Welfare Act, not designed to carry out medical procedures;
(2). veterinary clinical trials required for the marketing authorisation of a veterinary medicinal product carried out according to Article 37ah-37ak of the Act from September 6th 2001 – Pharmaceutical Law (Journal of Laws from 2008, No. 45, item 271 as amended in item 4);
(3). activity aimed at identifying animals;
(4). capturing wild animals for biometric and systematic assessment;
(5). veterinary procedures which do not cause pain, suffering, distress or permanent health impairment equal to or more invasive than the insertion of a needle.
(6). it is not a procedure to kill an animal solely to use its organs or tissues

Hence, according to point 1 described above, using peripheral blood, which is collected during routine veterinary procedures its retrieval and examination as described in paper titled: “New variants in ACE gene region in 10 Cavalier King Charles Spaniel dogs with mild and severe myxomatous mitral valve disease – preliminary study” did not require the approval of the Ethics Committee.

## 3 Results

During the recruitment period, 50 CKCS dogs were examined, 10 of which met the inclusion criteria. Five dogs were in stage B1, four dogs in stage C, and one dog in stage D (Table 1).

**Table 1:** List of enrolled dogs with ACVIM stage, age and gender specified.

ID	AN03	AN08	AN11	AN15	AN21	AN17	AN25	AN27	AN31	AN14
ACVIM STAGE	B1	B1	B1	B1	B1	C	C	C	C	D
AGE	8	9	7	7	7	7	10	8	6	7
SEX	M	F	F	F	M	M	F	F	F	M

Targeted NGS of the ACE gene achieved adequate coverage in all samples. Variant calling and downstream filtering yielded a total of 188 distinct sequence variants across the analyzed ACE genomic region, encompassing coding exons and flanking intronic sequences. All but one detected variant passed the standard PASS filter, one low-frequency intronic variant with a QD2 flag was retained.

Among the 188 detected variants, the overwhelming majority were located in non-coding regions. Overall, 183/188 variants (97.3%) were non-coding, including intronic, upstream, and 3′ UTR variants. Functional annotation showed that 179/188 variants (95.2%) carried an intron_variant annotation, of which 176/188 (93.6%) were annotated exclusively as intron_variant. Upstream gene variants accounted for 3/188 variants (1.6%), while 3′ UTR variants accounted for 1/188 variant (0.5%). Coding-region consequences were rare and included three synonymous variants (3/188, 1.6%), one missense variant (1/188, 0.5%), and one inframe insertion (1/188, 0.5%). Four variants (4/188, 2.1%) were annotated as splice-related, including splice region, splice donor region, and/or splice polypyrimidine tract variants. These categories are partially overlapping because several splice-related variants were also annotated as intronic or synonymous.

Of the detected variants, 174 already exist in available databases (Genbank, Ensembl, European variation archive), while 14 variants are unique to the studied population and have not been previously described [Table 2.]. All of these variants were located within intronic regions of the ACE gene and were annotated as intron variants with modifier impact.

**Table 2:** Summary of undescribed variants in the ACE gene region, divided into mild and severe MMVD groups. REF-reference, ALT - alternative.

Position on chromosome 9; ref. sequence NC_051813.1	REF	ALT	Total count alt alleles	Mild MMVD allele count	Severe MMVD allele count	Mild MMVD HET count	Severe MMVD HET count	Mild MMVD HOM count	Severe MMVD HOM count
13162573	TAA	TA (1),T (2),	10	4	6	4	2	0	2
13170911	G	GT (1),GTT (2),	11	5	6	5	2	0	2
13179224	TCC	TC (1),T (2),	13	5	8	4	4	0	1
13181013	G	GA	1	1	0	1	0	0	0
13182104	C	CTTTA (1),CTTTATTTA (2),CTTTATTTATTTA (3),	15	7	8	5	3	0	2
13187438	CGGG	C	4	0	4	0	0	0	2
13187689	TG	T (1),TGG (2),	10	4	6	3	2	0	2
13191266	T	TCCAAGAATAAA	1	1	0	1	0	0	0
13191267	C	CCAAGAATAAAA	11	5	6	5	2	0	2
13193678	C	G	7	4	3	4	3	0	0
13193686	C	G	8	3	5	3	3	0	1
13193690	C	G	3	3	0	3	0	0	0
13196108	G	GATTTATTT (1),GATTTATTTATTT (2),GATTT (3),	15	7	8	5	3	0	2
13196281	C	A	1	0	1	0	1	0	0

Intronic variants collectively carried the highest allelic burden, with a total of 1342 alternative alleles observed across the cohort (680 in mild and 662 in severe MMVD dogs). In contrast, non-intronic categories contributed substantially fewer alternative alleles. Upstream variants accounted for 4 alternative alleles (all in mild dogs), while the 3′ UTR variant contributed 12 alleles (6 mild and 6 severe). Coding sequence changes were relatively uncommon and included 27 synonymous alleles (15 in mild group and 12 in severe group), a single inframe insertion totaling 10 alternative alleles (4 in mild group and 6 severe group), and 3 alleles of a missense variant, all detected in mild group dogs. Variants affecting splice-associated regions collectively accounted for 32 alternative alleles (15 in mild group and 18 in severe group of dogs), all splice-related annotations, including splice region, splice donor region, and splice polypyrimidine tract variants, were considered.

Analysis of the variant frequencies in the studied dataset showed that the most frequently observed polymorphism in the ACE gene was the variant located at position 9:13,179,374 G>A (REF: G, ALT: A), which was characterized by the highest total number of alternative alleles in the entire analyzed cohort (Total_count_alt_alleles = 20). This variant has already been detected and is designated with the ID number rs3333762871.

The second most frequent variant was at position 9:13,193,659 with a total number of alternative alleles of 19 (10 in mild group, 9 in severe group). This variant has already been detected and is designated with the ID number rs7110130821

In the analyzed dataset, the variant rs850683722 located at position 9:13171340 corresponds to an A>G nucleotide substitution and is classified as an intronic variant (intron_variant, intron 15/25) with a predicted functional impact of MODIFIER. This variant does not result in any change to the protein-coding sequence (no HGVSp annotation), and its transcript-level description (c.2333-562A>G) indicates that it is positioned deep within the intron, at a considerable distance from exon-intron boundaries.

The variant was detected at a relatively high frequency in the studied cohort, with a total of 12 alternative alleles, and was present in both dogs with a mild course of disease (6 alternative alleles) and those with severe course of disease (6 alternative alleles). No statistically significant association was observed for MMVD severity or sex. The variant showed identical allelic distribution between Mild and Severe dogs (ALT/REF: 6/4 vs 6/4; Fisher’s exact test, p = 1.000) and no significant sex-related difference (p = 0.3729) In both subgroups, the variant occurred in heterozygous as well as homozygous alternative genotypes [Table 3.] (Meurs et al., 2018, Meurs et al., 2018, Meurs et al., 2015, Adin et al., 2021, Meurs et al., 2017, Adin et al., 2020).

**Table 3:** Distribution of rs850683722 variant in the studied population. HET-heterozygote, HOM-homozygote GT-genotype.

POS	REF	ALT	Total count alt alleles	Mild MMVD count alt alleles	Severe MMVD count alt alleles	Mild MMVD count HET	Severe MMVD count HET	Mild MMVD count HOM	Severe MMVD count HOM	AN03_GT	AN08_GT	AN11_GT	AN15_GT	AN21_GT	AN14_GT	AN17_GT	AN25_GT	AN27_GT	AN31_GT
13171340	A	G	12	6	6	4	2	1	2	0/1	0/1	0/1	1/1	0/1	1/1	1/1	0/1	0/0	0/1

The single missense variant was located in position 9:13198399 A>C in the coding region and resulted in a Glu1300Asp amino acid substitution with a predicted functional impact of MODERATE.

The inframe insertion (p.Pro18_Leu19dup) represented a duplication of two amino acids in the N-terminal portion of the protein and was observed with 10 alternative alleles (4 in mild MMVD and 6 in severe MMVD dogs). This variant is already existing in genetic databases with ID rs3333622365 and has predicted functional impact of MODERATE. No in silico pathogenicity prediction was available for this insertion.

Splice-region-associated variants together accounted for 32 alternative alleles (14 in mild group and 18 in severe group of dogs). One of these variants was concurrently annotated as synonymous (p.Gly292=), whereas the remaining changes were located within the splice donor region or polypyrimidine tract adjacent to intronic boundaries.

None of the analysed variants showed a statistically significant association with disease severity or gender, either in the allelic model or in the carrier model. No variant reached nominal significance at p < 0.05. The lowest p-value in the allelic analysis was observed for rs7110131114 variant at position 9:13,187,449, for which alternative alleles were detected in the mild group but not in the severe group; however, this result did not reach statistical significance (Fisher’s exact test, p = 0.0606). In the carrier-based analysis, the same variant also showed the lowest p-value, but again without statistical significance (p = 0.1000). In the gender allelic model, the lowest p-value obtained was p = 0.1698 for any variant.

## 4 Discussion

The ACE gene in dogs is a key component of the RAAS. However, depending on the reference genome used, its structural and genomic description is subject to significant modifications. In the newer reference genome ROS_Cfam_1.0 (2020) compared to CanFam3.1(2011), based on a chromosomal assembly with significantly higher contiguity, the basic architecture of the gene (the number of exons and introns) is preserved, but the chromosomal coordinates, total gene length, and detailed transcript annotation are subject to adjustments. Importantly, a change in the reference genome can lead to a reversal of the reference and alternative alleles for the same SNP variants within ACE and other genes, despite the biological invariance of the polymorphism itself. Therefore, in comparative analyses and interpretation of genetic data regarding ACE, it is crucial to clearly indicate the version of the reference genome used, the annotation system, and the method of variant normalization. Taking into account possible allele changes of individual SNPs in relation to the older and current reference genome, the previously mentioned rs850683722 variant cannot be omitted (Meurs et al., 2018, Meurs et al., 2015, Adin et al., 2021, Meurs et al., 2017, Adin et al., 2020). The ideal situation would be to use a breed-specific reference genome, however to the best of our current knowledge, no reference genome is available for the CKCS.

While the clearly demonstrated association of the presence of variants within the genes encoding RAAS elements was reflected in humans, our results with an exceptionally high number of variants only in the ACE gene region without clear differentiation between individuals indicate the complexity of the problem and the need for cautious conclusions on this subject (Meurs et al., 2018, Ozisik et al., 2004, Cheng et al., 2025, Han et al., 2017).

The decision to select a 6-year age limit for the dogs selected for the study was based on population studies reporting the occurrence of murmurs in approximately 50% of dogs at this age. We aimed to obtain a group of dogs as young as possible, with healthy dogs selected as the reference group, and those in the most advanced stages of the disease as the study group. Due to the lack of healthy dogs above this age in the obtained population, we decided to select stage B1 as the reference group (Lundin and Kvart, 2010, Birkegård et al., 2016).

The majority of the 14 novel loci were detected in both clinical groups, although the number of alternate alleles varied between dogs with mild and severe MMVD. Several variants were present in both groups with comparable allele counts (positions 9:13,162,573; 13,170,911; 13,179,224; 13,182,104; 13,187,689; 13,191,267; 13,193,678; 13,193,686; and 13,196,108), suggesting that they represent background polymorphisms within the ACE locus rather than stage-specific variants.

A smaller subset of variants showed stage-restricted occurrence in this cohort. Variants detected only in dogs with severe MMVD were located at positions (9:13,187,438; 13,196,281), whereas variants observed exclusively in dogs with mild disease occurred at positions (9:13,181,013; 13,191,266; 13,193,690). However, these loci were detected in a very limited number of individuals, and therefore their apparent stage-specific distribution should be interpreted cautiously.

An interesting observation is that in the group with mild MMVD no homozygotes for previously non-described alternative alleles of any variant were detected, which requires further analyses in the future on larger groups of animals.

The rs850683722 variant was found in both groups with both homozygotic and heterozygotic individuals. Also in the context of this variant, no statistically significant differences were detected between the groups, and the distribution of alleles was equal. The aim of this study, in the context of this specific variant, was solely to assess the occurrence of this variant in the mild and severe MMVD groups. Given that previously published studies have assessed this variant in terms of its effect on RAAS activity and the efficacy of its inhibition, the next step should be to analyze its effect on the course of MMVD in a large population-based study (Meurs et al., 2018, Meurs et al., 2015, Adin et al., 2021, Meurs et al., 2017, Adin et al., 2020). An important point in the discussion of the significance of this variant and its impact is also to consider the reversal of the reference and alternative alleles along with the change in the dog reference genome. It is important to emphasize that K. Meurs's work describes the current reference variant as an alternative variant that, at the time of publication, referenced a different reference genome (Meurs et al., 2018, Meurs et al., 2015, Adin et al., 2021, Meurs et al., 2017, Adin et al., 2020).

Considering the rs7110130821 and rs3333762871 variants and their occurrence in all dogs despite the presumed lack of relationship between dogs, they should be considered as potential reference variants for the CKCS breed with future evaluation against other dog breeds.

Referring to a single detected missense with ID of rs8601782 in the coding fragment of the ACE gene. In silico prediction (SIFT score 0.32, “tolerated – low confidence”) together with a modest BLOSUM62 score (2) indicates that this substitution is likely to be functionally conservative rather than strongly deleterious. Moreover, this variant was detected in only three alternative alleles, all in dogs with early-stage MMVD, and was absent from the late-stage group, arguing against a major role as a driver of severe disease in this cohort. No literature describing the possible effect of this variant on RAAS activity was found.

Although inframe insertions may theoretically alter local structure or processing of the protein, the balanced distribution of rs3333622365 variant between mild and severe MMVD groups and its moderate overall frequency suggest that any functional effect, if present, is likely to be subtle.

Considering variants in splicing regions, while such variants have theoretical potential to modulate splicing efficiency or exon inclusion, their low frequency and symmetric distribution between disease stages in this dataset do not support a strong association with MMVD severity (Anna and Monika, 2018).

Deep intronic variants (DIVs), typically located more than ∼100 bp from exon–intron boundaries, can significantly influence gene expression by disrupting regulatory mechanisms involved in pre-mRNA processing. These variants most commonly create or strengthen cryptic splice sites, leading to the inclusion of intronic fragments as pseudoexons in the mature transcript. Such aberrant splicing events may introduce premature termination codons, resulting in truncated proteins or degradation of the transcript via the nonsense-mediated decay pathway. In human medicine, deep intronic mutations have been implicated in numerous monogenic disorders; for example, intronic variants in the CFTR gene can activate cryptic splice sites and alter transcript structure in cystic fibrosis. (Anna and Monika, 2018)

Several limitations of this study should be acknowledged. First, the sample size limits statistical power, particularly for the detection of low-frequency coding variants and for robust association testing between genotype and clinical phenotype. Second, the analysis was restricted to sequence variation and did not incorporate gene expression data or direct measurements of ACE activity, which would be necessary to establish functional relevance. Third, population stratification and breed-specific effects could influence allele frequencies and were not fully addressed in the current dataset. Given the known breed predispositions for MMVD, future studies should explicitly integrate breed structure into genetic analyses.

Taken together, the ACE variant spectrum in this CKCS cohort is characterized by a high density of non-coding, MODIFIER-impact intronic variants, accompanied by a small number of rare coding and splice-region variants with LOW to MODERATE predicted impact and no clear segregation with clinical severity. These findings support the interpretation that, beyond the intronic variant with the highest allelic burden previously analyzed in detail, most additional ACE polymorphisms in this dataset are unlikely to act as primary determinants of MMVD progression, although subtle regulatory or modifier effects - especially for the missense, inframe insertion, and splice-region variants - cannot be entirely excluded. An additional factor supporting this thesis is the previously described lack of expression of angiotensin II receptors and angiotensin-converting enzyme in degenerative valves in dogs (Mow and Pedersen, 1999). The association of genetic variation in genes determining RAAS components with the different course of later stages of MMVD requires further analysis.

To the best of our knowledge this study is the first to analyze the full sequence of the entire ACE gene region in CKCS using NGS, providing a significant contribution to the understanding of the ACE gene in this breed. Previously published studies have tended to focus on single loci, from which variants of potential clinical significance have been identified (Meurs et al., 2018, Meurs et al., 2015, Adin et al., 2021, Meurs et al., 2017, Adin et al., 2020).

In addition, two variants were identified in the analyzed population that may be potentially specific to this breed, but this requires further validation in larger cohorts of dogs.

In conclusion, our results indicate that genetic variation within the canine ACE gene is predominantly non-coding and that differences between mild and severe MMVD are subtle and regulatory in nature. While no single variant emerged as a strong predictor of disease severity, the observed distribution of intronic variants supports a modulatory role for ACE in MMVD progression, consistent with its central position within the RAAS. These findings emphasize the importance of considering regulatory genetic variation and polygenic interactions when investigating the molecular basis of complex cardiovascular diseases in dogs.

## 5 Ethics approval statement

In accordance with the Experiments on Animals Act from January 15th 2015 (Journal of Laws of the Republic of Poland, 2015, item. 266), concerning the welfare of the animals used for research or teaching purposes, the restrictions defined in the law shall not apply to:

(1). clinical veterinary practices as defined by the Act from December 18th 2003 concerning veterinary practices (Journal of Laws from 2004, No. 11, item 95 as amended in item 3), as well as agricultural activity, raising and breeding livestock according to the Animal Welfare Act, not designed to carry out medical procedures;
(2). veterinary clinical trials required for the marketing authorisation of a veterinary medicinal product carried out according to Article 37ah-37ak of the Act from September 6th 2001 – Pharmaceutical Law (Journal of Laws from 2008, No. 45, item 271 as amended in item 4);
(3). activity aimed at identifying animals;
(4). capturing wild animals for biometric and systematic assessment;
(5). veterinary procedures which do not cause pain, suffering, distress or permanent health impairment equal to or more invasive than the insertion of a needle.
(6). it is not a procedure to kill an animal solely to use its organs or tissues

Hence, according to point 1 described above, using peripheral blood, which is collected during routine veterinary procedures its retrieval and examination as described in paper titled: “New variants in ACE gene region in 10 Cavalier King Charles Spaniel dogs with mild and severe myxomatous mitral valve disease – preliminary study” did not require the approval of the Ethics Committee.

## CRediT authorship contribution statement

**Maksymilian Lewicki:** Writing – review & editing, Writing – original draft, Software, Investigation, Funding acquisition, Formal analysis, Data curation, Conceptualization. **Sylwia Górczyńska-Kosiorz:** Writing – review & editing, Writing – original draft, Validation, Supervision, Methodology, Investigation, Conceptualization. **Piotr Frydrychowski:** Writing – review & editing, Methodology, Investigation, Funding acquisition, Formal analysis. **Justyn Gach:** Writing – review & editing, Writing – original draft, Methodology. **Zuzanna Wojtczak:** Writing – review & editing, Software, Investigation. **Jacek Cymbryłowicz:** Writing – review & editing, Validation, Supervision, Investigation. **Agnieszka Noszczyk-Nowak:** Writing – review & editing, Validation, Supervision, Resources, Project administration, Methodology, Investigation, Funding acquisition, Conceptualization.

## Declaration of competing interest

The authors declare the following financial interests/personal relationships which may be considered as potential competing interests:

Maksymilian Lewicki reports financial support was provided by Republic of Poland Ministry of Science and Higher Education. If there are other authors, they declare that they have no known competing financial interests or personal relationships that could have appeared to influence the work reported in this paper.

## Data Availability

All relevant data are available in the manuscript text and supplementary material.

## Appendix Supplementary materials

Image, application 1
